# Brassinosteroid preharvest treatments as a useful tool to increase crop yield and red colour in blood orange fruits

**DOI:** 10.3389/fpls.2025.1654517

**Published:** 2025-09-02

**Authors:** Fernando Garrido-Auñón, Jenifer Puente-Moreno, María E. García-Pastor, Pedro J. Zapata, Domingo Martínez-Romero, María Serrano, Daniel Valero

**Affiliations:** ^1^ Department of Agrofood Technology, Escuela Politécnica Superior de Orihuela (EPSO), Instituto de Investigación e Innovación Agroalimentario y Agroambiental (CIAGRO), University Miguel Hernández, Alicante, Spain; ^2^ Department of Applied Biology, Escuela Politécnica Superior de Orihuela (EPSO), Instituto de Investigación e Innovación Agroalimentario y Agroambiental (CIAGRO), University Miguel Hernández, Alicante, Spain

**Keywords:** *Citrus sinensis*, 24-epibrassinolide, anthocyanin, commercial quality, elicitation, phenolics, soluble solids, acidity

## Abstract

**Introduction:**

Blood oranges are highly appreciated by consumers due to their attractive peel, red-colour juice, and their antioxidant and other health-beneficial properties. However, increased temperatures due to climate change compromise anthocyanin biosynthesis, depreciating their market value. Brassinosteroids (BRs) are plant hormones with important effects in plant adaptation to environmental stresses.

**Materials and methods:**

The present experiment aimed to evaluate the effects of tree foliar treatments with different 24-epibrassinolide (24-BL) concentrations (0.01, 0.1, and 1 μM) on crop yield and fruit quality properties of the “Sanguinelli” blood oranges, with special emphasis on anthocyanin content in the peel and juice.

**Results:**

The results of 2-year experiments (2021–2022 and 2022–2023, carried out in Alicante, Spain) showed that 24-BL treatments improved total crop yield. Fruit quality properties, such as firmness, total soluble solids, and titratable acidity, were also enhanced, as well as red colour in the flavedo and juice, due to enhanced total and individual anthocyanin concentrations.

**Discussion:**

The greatest improvements were recorded at the 0.1-μM 24-BL treatment, in which the enhanced peel colour led to a 41% increase in the yield of commercial fruits compared to control trees, which could have an important beneficial impact on growers’ profit. In addition, the consumer acceptance of 24-BL could increase since the red colour in blood oranges is the most important parameter valued by consumers. With the enhancement of their total anthocyanin and phenolic contents, their health-beneficial effects are also increased.

## 1 Introduction

Blood orange is one of four groups within the sweet orange species (*Citrus sinensis* L. Osbeck) and is grown in a few areas of the Mediterranean Basin, including Spain and Italy (Habibi et al., 2022a). Spain is the main citrus-producing country in the European Union and the sixth in the world. However, citrus fruit production has decreased in the last two seasons in Spain, mainly due to unfavourable climatic change events (MAPA, 2024), and blood oranges represent only 1.1% of the total planted area of citrus fruits in Spain, the most important grown cultivar being “Sanguinelli”, which appeared as a spontaneous mutation of cv. Doble Fina in the region of Almenara (Castellón) in 1929 (MAPA, 2024). The main difference between blood orange fruits and other orange cultivars is the red colouration in the flesh and peel caused by the synthesis of anthocyanin pigments (Habibi et al., 2021; 2022a). Anthocyanins have been reported as the major component responsible for the antioxidant properties of blood oranges, together with other phenolic compounds (hydroxycinnamic acids and other flavonoids) and ascorbic acid, which are found at higher levels compared with yellow oranges (Morales et al., 2019; Legua et al., 2021; Habibi et al., 2022b; 2023). These compounds make the blood orange fruit one of the most valuable citrus fruits with health-related properties due to their effects in reducing the incidence and evolution of some human diseases, including cancer, arteriosclerosis, and cardiovascular diseases (Grosso et al., 2013; Fallico et al., 2017; Chen et al., 2024).

Moreover, anthocyanin content has been considered an important quality trait for blood orange commercialisation and is responsible for its increased consumption due to its attractive colour for consumers (Rapisarda et al., 2001; Habibi et al., 2022a). It has been demonstrated that the pigmentation process in blood orange fruits is dependent on cultivar, scion/rootstock combination, and environmental conditions, such as light and temperature differences between night and day, since larger temperature differences lead to an increase in the concentration of total anthocyanins (Butelli et al., 2012; Continella et al., 2018). However, the semi-arid climate area with increased temperatures in Spain has expanded by more than 30,000 km^2^ in the last decades, according to data from the Spanish Government (MITECO, 2024), affecting the southeast of Spain, the major area of blood orange production. Blood oranges grown under these climatic conditions have poor pigmentation, and their commercial value is negatively affected (Habibi et al., 2022a). Therefore, it is necessary to find new crop strategies or treatments to overcome this problem using eco-friendly and safe tools, ensuring safety and environmental responsibilities within the agricultural sector. Accordingly, preharvest or postharvest treatments with methyl jasmonate, salicylic acid, methyl salicylate, or γ-aminobutyric acid (GABA) have been reported to increase red colour, anthocyanins, and other health-promoting compounds in “Moro” blood oranges after cold storage (Habibi et al., 2020; Vithana et al., 2024). However, few methods are currently known to improve red colour pigmentation of the most important blood oranges in Spain.

In addition, brassinosteroids (BRs) are nowadays regarded as the sixth group of plant hormones with effects on growth, ripening, and quality properties of fleshy fruits when applied as preharvest or postharvest treatments (Garrido-Auñón et al., 2024; Gutiérrez-Villamil et al., 2024). Moreover, BRs have been reported as potential compounds to protect plants from abiotic (heat, drought, or flooding) and biotic stresses, with positive effects on enhancing crop yield in cereals, legumes, and even fresh fruit species and, in turn, overcoming the detrimental effects of stress on agricultural profits (Ali, 2017; Manghwar et al., 2022; Khan et al., 2023; Garrido-Auñón et al., 2024; Silva et al., 2024). Most of the experiments reported in the previous literature have been performed with brassinolide (BL) and 24-epibrassinolide (24-BL), which are considered to be among the most active BRs in plants (Garrido-Auñón et al., 2024; Lateef et al., 2023). Nevertheless, the observed effects were dependent on fruit species, concentrations, and time of application, and only a few studies have addressed the use of BRs as potential elicitors in preharvest to improve crop yield and fruit quality traits at harvest. For instance, Mostafa and Kotb (2018) showed that spraying BL at a concentration of 1.0 mg/L increased fruit set percentage, fruit retention, number of fruits per tree, and yield in sugar apple trees. However, the use of 24-BL in a concentration of 0.1 mg/L was enough to obtain higher fruit production and better quality attributes in strawberries compared to the controls (Furio et al., 2022). Accordingly, other researchers have highlighted the use of BRs as a preharvest potential tool to improve yield and fruit quality traits at harvest in strawberries and grapes cv. Alphonse Lavallée (Babalık et al., 2020; Khatoon et al., 2020). Moreover, treatment with BRs has been reported to improve anthocyanin content in strawberries (Ayub et al., 2018) and different red table grape cultivars (Zhou et al., 2018; Vergara et al., 2020). Specifically, in “Sanguinello” blood oranges, it has been reported that postharvest treatments with 24-BL increased total anthocyanin content and reduced symptoms of chilling injury during cold storage (Habibi et al., 2021).

Based on some literature (Furio et al., 2022; Babalık et al., 2020; Khatoon et al., 2020), it was hypothesised that the tree 24-BL treatment could be a new eco-friendly tool with positive impacts on enhancing crop yield and fruit quality traits, particularly red pigmentation in the fruit peel and juice, of “Sanguinelli” blood orange trees, the most important cultivar grown in Spain. Then, the 24-BL application could mitigate the negative impact of the expansion of semi-arid climatic conditions in the Spanish production area of blood oranges on anthocyanin biosynthesis, enhancing the commercial value of this citrus fruit cultivar and satisfying consumer demands since external red colouration is the main quality parameter in blood oranges that is highly appreciated by consumers. As far as we know, this is a new approach since the role of BRs as a preharvest elicitor treatment in blood orange trees to increase crop yield, anthocyanin content, and other fruit quality traits has never been studied. Therefore, this study focused on evaluating the effects of 24-BL applied as a foliar spray, at different concentrations (0.01, 0.1, and 1 μM), on crop yield and fruit quality properties of “Sanguinelli” blood orange fruits, with special emphasis on anthocyanin content in the peel and juice. The experiments were conducted over two growing seasons, and the expected results could lead to an improved market value of this blood orange cultivar by overcoming the negative impact of semi-arid climatic conditions on crop yield and anthocyanin biosynthesis.

## 2 Materials and methods

### 2.1 Plant materials and experimental design

The research was carried out in a commercial plot (from the company, Las Moreras Fruits & Veggies) located at Algorfa (Alicante, Spain, GPS coordinates 38.08369, −0.77392). Blood orange trees (*C. sinensis* L.) of the “Sanguinelli” cultivar were grafted onto “Macrophyla” rootstock in 2011, and the experiments were carried out from October to February in two different seasons, 2021–2022 and 2022–2023, with trees being 10 and 11 years old. In both seasons, a randomised block experimental design was set with three blocks of three trees for each treatment. In the first season, 0.01-, 0.1-, and 1-μM 24-BL concentrations were used, and in the second season, 0.01- and 0.1-μM concentrations were used. In the first season, all treatments improved total crop yield, yield of commercial fruits, and fruit quality traits, with the effects being, in general, dose dependent from 0.01 to 0.1 μM, while the highest 24-BL concentration (1 μM) did not improve the results obtained with 0.1 μM. Thus, 0.01 and 0.1 μM doses were applied in the second season in order to prove the repeatability of the results obtained during the first year, while the highest dose was not used because, in practical applications, lower doses could be more cost-effective than higher doses in obtaining similar results. 24-BL was purchased from Sigma (Sigma-Aldrich, Madrid, Spain; CAS Number: 72962-43-7). The calculated amount of 24-BL to prepare the desired concentration was dissolved in 15 mL of ethanol and, thereafter, distilled water was added up to 13.5 L, with 13.5 mL of Elogium^®^ (Alquil-Poliglicol Ether 20% w/v, SIPCAM Iberia, Valencia, Spain) as surfactant. Preharvest treatments were performed by foliar spray application of 1.5 L per tree of the corresponding 24-BL concentration using a mechanical mist sprayer, which was sufficient to wet the entire tree canopy to the point of drip, according to previous experiments carried out on several tree species such as sweet cherries (*Prunus avium*), pomegranates (*Punica granatum*), lemons (*Citrus lemon*), and blood oranges (*C. sinensis*) (Guirao et al., 2025; Badiche-El Hilali et al., 2023; 2024; Carrión-Antolí et al., 2024; Lorente-Mento et al., 2023). Each 24-BL dose was applied three times at three key points of fruit development according to the colour changes in peel and juice vesicles and the results of the study by Guirao et al. (2025): T1, at the beginning of flavedo degreening, when chlorophyll started to disappear; T2, when the red colour appeared at the fruit stylar end; and T3, 3 days before harvest (**Table 1**). Control trees were sprayed with distilled water containing 1 mL/L of Elogium^®^. All trees were grown under similar environmental conditions and agricultural practices for the blood orange crop. Trees were sprayed carefully without leaf damage, and no negative effects on the tree performance and continuous production capacity were observed as a consequence of the 24-BL treatments.

**Table 1:** Treatment dates with 24-epibrassinolide (T1, T2, and T3)* and harvest dates of “Sanguinelli” blood oranges for the two different seasons.

Season	T1	T2	T3	Harvest
2021–2022	13/10/2021	13/12/2021	05/02/2022	08/02/2022
2022–2023	3/10/2022	12/12/2022	22/01/2023	25/01/2023
	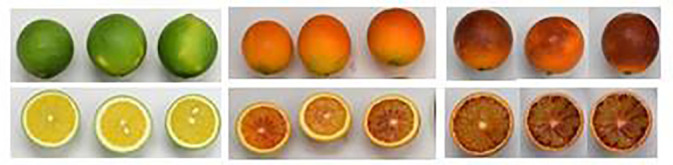			

*Photographs show the external and internal fruit appearance for T1, T2, and T3 treatments.

### 2.2 Determination of crop yield

Fruits were harvested when most of the fruits on the tree reached their commercial ripening stage based on fruit size and flavedo red colour (**Table 1**), that is to say, with fruit diameter higher than 65 mm and ca. 50% of fruit surface showing red colour. Then, all fruits harvested from each tree were weighed and counted in the field to obtain crop yield (in kg/tree and number of fruits/tree), and the average fruit weight of each tree was calculated. In the 2021–2022 experiment, all the harvested fruits from the nine trees of each treatment were transported to Las Moreras Fruits & Veggies facilities and visually separated into commercial and non-commercial fruits, according to the commercial criteria for this blood orange cultivar, as follows: commercial fruits having red colour in more than 25% of their surface and non-commercial fruits having red colour in less than 25% of their surface. In addition, commercial and non-commercial fruits were classified into four size categories based on equatorial diameters of 77–73, 73–70, 70–67, and 67–64 mm. Fruit classification according to size was performed using an ORHEA II V2.0 OPTISCAN II machine (MAF RODA, RODA IBÉRICA, Alzira, Valencia, Spain). Finally, four replicates of five fruits per treatment, homogenous in size and colour and without visual defects, were selected at random from the second category of commercial fruits (73–70 mm in diameter) and transported to the laboratory within 60 min, where the following analytical determinations were performed.

### 2.3 Fruit quality parameters

Fruit firmness was determined in each fruit as a force–deformation ratio (N/mm) using a TA-XT2i Texture Analyser (Stable Microsystems, Godalming, UK). The texture analyser was equipped with a steel flat plate device, which, after measuring the fruit diameter, applied a force to achieve a 5% deformation of the fruit equatorial diameter. External colour was measured at six equidistant points of the equatorial fruit perimeter using a Minolta colourimeter (CRC400; Minolta, Osaka, Japan) to record the L*, a*, and b* coordinates in the CIE Lab system. Then, the fruits were cut by their equatorial diameter, and internal colour was measured at three equidistant points of the cut surface, avoiding the albedo tissue. For firmness and colour, data are presented as the mean ± SD of four replicates, each one composed of five fruits.

Then, orange juice was obtained using an electrical squeezer, and the juice obtained from the five fruits of each replicate was combined to obtain four biological samples in which the following parameters were measured. Similarly, four samples were obtained by combining the flavedo of the five fruits of each replicate, which were freeze-dried, ground, and stored at −80°C until anthocyanins and phenolics were measured. Total soluble solids (TSS) were measured in duplicate in each juice sample using a digital refractometer (model Atago PR-101, Atago Co., Ltd., Tokyo, Japan) at 20°C, and the results (mean ± SD) were expressed as grams per 100 g. Titratable acidity (TA) was also determined in duplicate in each juice sample by automatic titration (785 DMP Titrino; Metrohm, Herisau, Switzerland), with 0.1 N NaOH and pH reaching 8.1, using 1 mL of juice in 25 mL of distilled water. The results (mean ± SD) were expressed as grams of citric acid equivalent per 100 ^—^g fresh weight. Finally, for each replicate, 15 mL of juice was frozen at −20°C until anthocyanins and phenolics were quantified.

### 2.4 Quantification of total and individual anthocyanins and total phenolics

Flavedo and juice anthocyanin extractions and quantifications were carried out, adapting the method reported in Habibi et al. (2021). For the flavedo, 0.2 g of freeze-dried powder was mixed with 5 mL of methanol/hydrochloric acid/water (25:1:24, v/v/v) and was extracted using a mortar and pestle. The extracts were centrifuged at 10,000 × *g* for 10 min at 4°C. Finally, total anthocyanin content was quantified in the supernatant by reading absorbance at 520 nm using a spectrophotometer (UNICAM Helios-α, Artisan Technology Group, Champaign, IL, USA), and the results were expressed as milligrams of cyanidin 3-*O*-glucoside equivalent per 100 g. Total anthocyanin content in the juice was measured similarly using 2.5 mL of juice in 5 mL of the extraction mixture. Thereafter, the supernatant from the juice was filtered through a 0.45-μm polyvinylidene fluoride (PVDF) filter (Millex-HV13, Millipore, Bedford, MA, USA), and quantification of individual anthocyanins was performed by high-performance liquid chromatography (HPLC) analysis as previously reported (García-Pastor et al., 2020). Briefly, the HPLC system was equipped with a Luna C18 column (25 cm × 0.46 cm i.d. and 5-μm particle size; Phenomenex, Macclesfield, UK) and a C18 column guard (4.0 mm × 3.0 mm) cartridge system (Phenomenex). The mobile phases were as follows: (A) water–formic acid (99:5, v/v) and (B) acetonitrile, running at a flow rate of 1 mL/min, with a linear gradient starting with 8% of solvent B, reaching 15% at 25 min, 22% at 55 min, and 40% at 60 min, which was maintained up to 70 min. The injection volume was 20 μL, and the chromatograms were recorded at 520 nm. Anthocyanin standards were cyanidin 3-(6-malonyl)-glucoside, cyanidin 3-*O*-glucoside, and peonidin 3-*O*-glucoside (purchased from Sigma-Aldrich, Schnelldorf, Germany). The results are the mean ± SD of measures performed in duplicate in four replicates.

Phenolic compounds were extracted by homogenising 0.2 g of flavedo or 5 mL of juice with 15 mL of water:methanol (2:8, v/v) containing 2.0-mM NaF (1.5 w/v) and centrifuged as indicated above. Then, 50 and 200 μL of the supernatant, for the juice and flavedo, respectively, were mixed with 500-μL phosphate buffer (50 mM, pH 7.8) and 2.5 mL of water-diluted Folin–Ciocalteau (1:10) reagent and incubated for 2.5 min at room temperature to carry out the colourimetric reaction (Díaz-Mula et al., 2009). Then, 2 mL of sodium carbonate (53 g/L) was added and shaken vigorously. Thereafter, the mixture was incubated in a water bath at 50°C for 5 min and, finally, the absorbance was measured at 760 nm, with the results expressed as milligrams of gallic acid equivalent per 100 g fresh weight (mean ± SD) using a calibration curve previously performed.

### 2.5 Statistical analysis

The experimental data for each season were subjected to one-way analysis of variance (ANOVA). Honestly significant difference (HSD) Duncan’s test was performed to determine significant differences among treatments at *p* < 0.05. All statistical analyses were performed using the SPSS v. 22.0 software package for Windows.

## 3 Results

### 3.1 Effect of 24-BL treatments on crop yield

**Figure 1:**
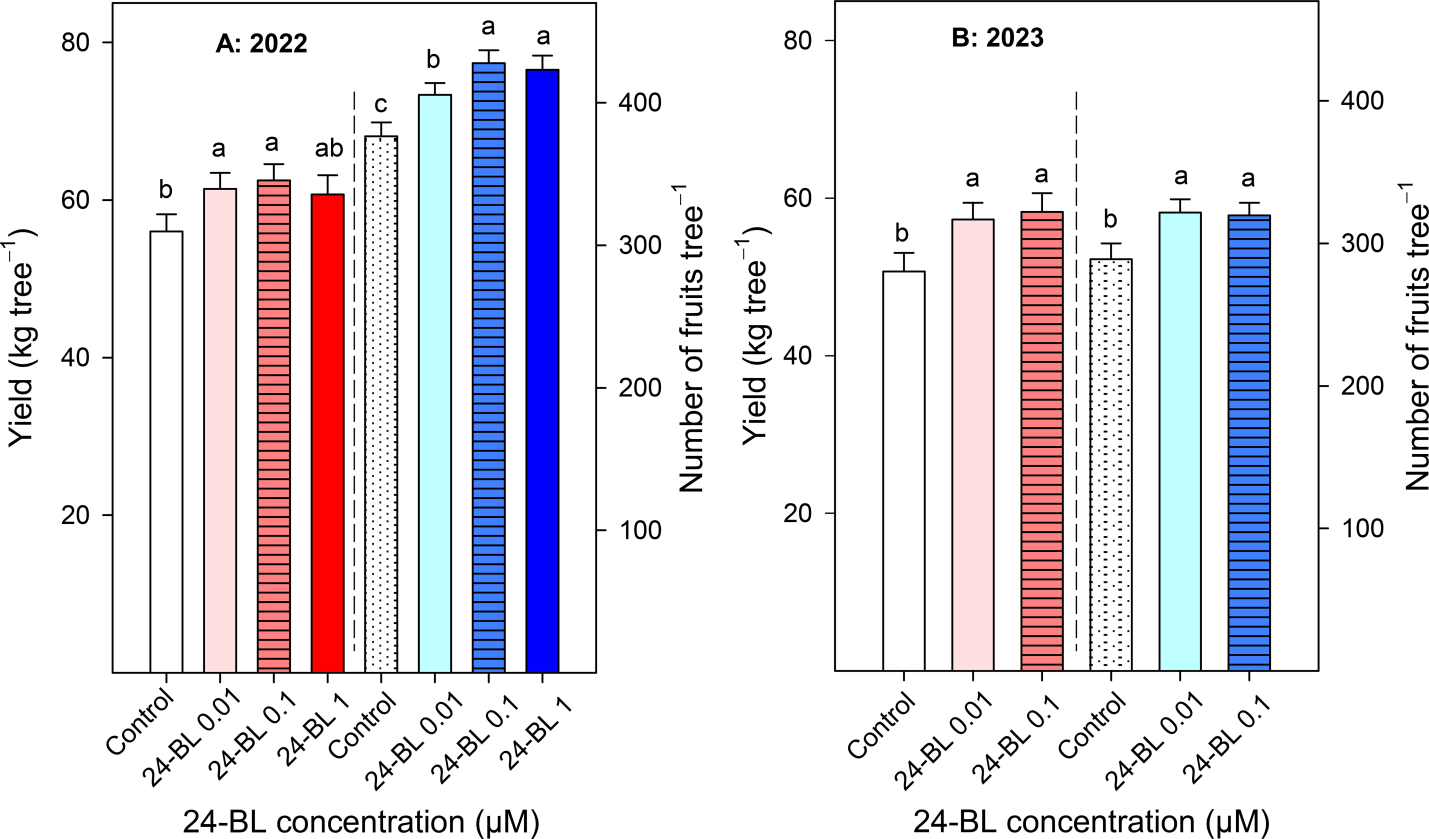
Blood orange yield (kg/tree, left axis; number of fruits/tree, right axis) in two different seasons, 2022 **(A)** and 2023 **(B)**, as affected by 24-epibrassinolide (24-BL) treatments at 0.01, 0.1, and 1 μM. Data are presented as the mean ± SD. Different letters show significant differences (*p* < 0.05) among treatments for each growing season.

In the 2021–2022 experiment, 24-BL at 0.01- and 0.1-μM concentrations (61.42 ± 2.04, 62.49 ± 2.06 kg/tree) significantly increased crop yield compared to the control trees (56.04 ± 2.18 kg/tree) (**Figure 1A**). However, the highest concentration at 1 μM did not show significant differences in the control or the other treatments. Similar results were obtained in the 2022–2023 experiment since crop yield was also higher in 0.01- and 0.1-μM 24-BL-treated trees (57.30 ± 2.12 and 58.26 ± 2.39 kg/tree, respectively) compared to the controls (50.70 ± 2.37 kg/tree), without significant differences in both 24-BL doses (**Figure 1B**). However, there was also a positive effect of treatments on the number of fruits harvested per tree in both seasons. Thus, in the first season, all 24-BL treatments significantly increased the number of harvested fruits (*p* < 0.05) compared to the control, and the highest effects were found for 0.1 and 1 μM doses (**Figure 1A**). However, in the second season, the 0.01 and 0.1 μM 24-BL treatments also led to significant increases in the number of fruits harvested per tree with respect to the controls, but no significant differences were observed in both doses (**Figure 1B**). Nevertheless, when fruit weight was calculated (in kg/tree or number of fruits/tree), no significant effects attributed to the 24-BL treatments were observed, with values of 145–150 and 175–180 g for the 2022 and 2023 seasons, respectively.

### 3.2 Commercial and non-commercial crop yield

**Figure 2:**
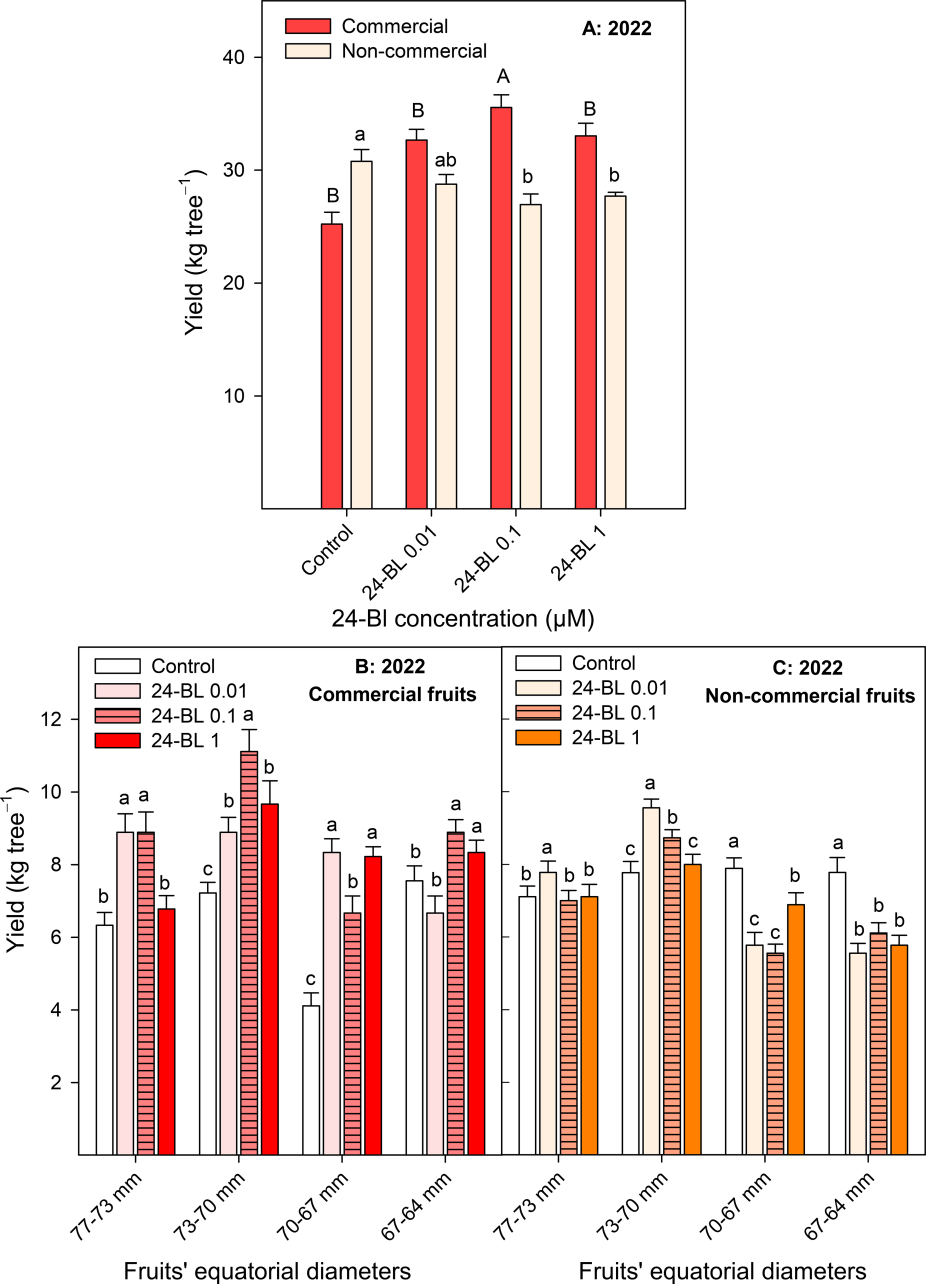
Effect of 24-epibrassinolide (24-BL) treatments, at 0.01, 0.1, and 1 μM, on total yield of commercial and non-commercial fruits **(A)** and distribution of commercial **(B)** and non-commercial **(C)** fruit yield on different categories depending on fruits’ equatorial diameters. Data are presented as the mean ± SD. Different capital and lowercase letters in panel A show significant differences (*p* < 0.05) among treatments for commercial and non-commercial fruits, respectively. Different lowercase letters in panels B and C show significant differences (*p* < 0.05) among treatments for each fruit category.

The results of the first growing season showed that all 24-BL treatments increased the yield of commercial fruits, with the highest effects being observed for 0.1 μM, with a total of 35.56 ± 1.10 kg/tree, compared to 25.22 ± 1.05 kg/tree of commercial fruits harvested from the control trees (**Figure 2A**). Consequently, the yield of non-commercial fruits was decreased by the treatments. Commercial and non-commercial fruits were classified into four size categories, according to equatorial diameters of 77–73, 73–70, 70–67, and 67–64 mm. The preharvest applications of 0.01-, 0.1-, and 1-μM 24-BL led to higher yields of commercial fruits with large sizes (77–73, 73–70, and 70–67 mm) than in the control trees (**Figure 2B**). In addition, for non-commercial blood orange fruits, the yield of large-sized fruits was also increased for 0.01- and 0.1-μM 24-BL, while the yield of small-sized fruits was lower in treated trees than in controls (**Figure 2C**).

### 3.3 Fruit quality parameters

**Figure 3:**
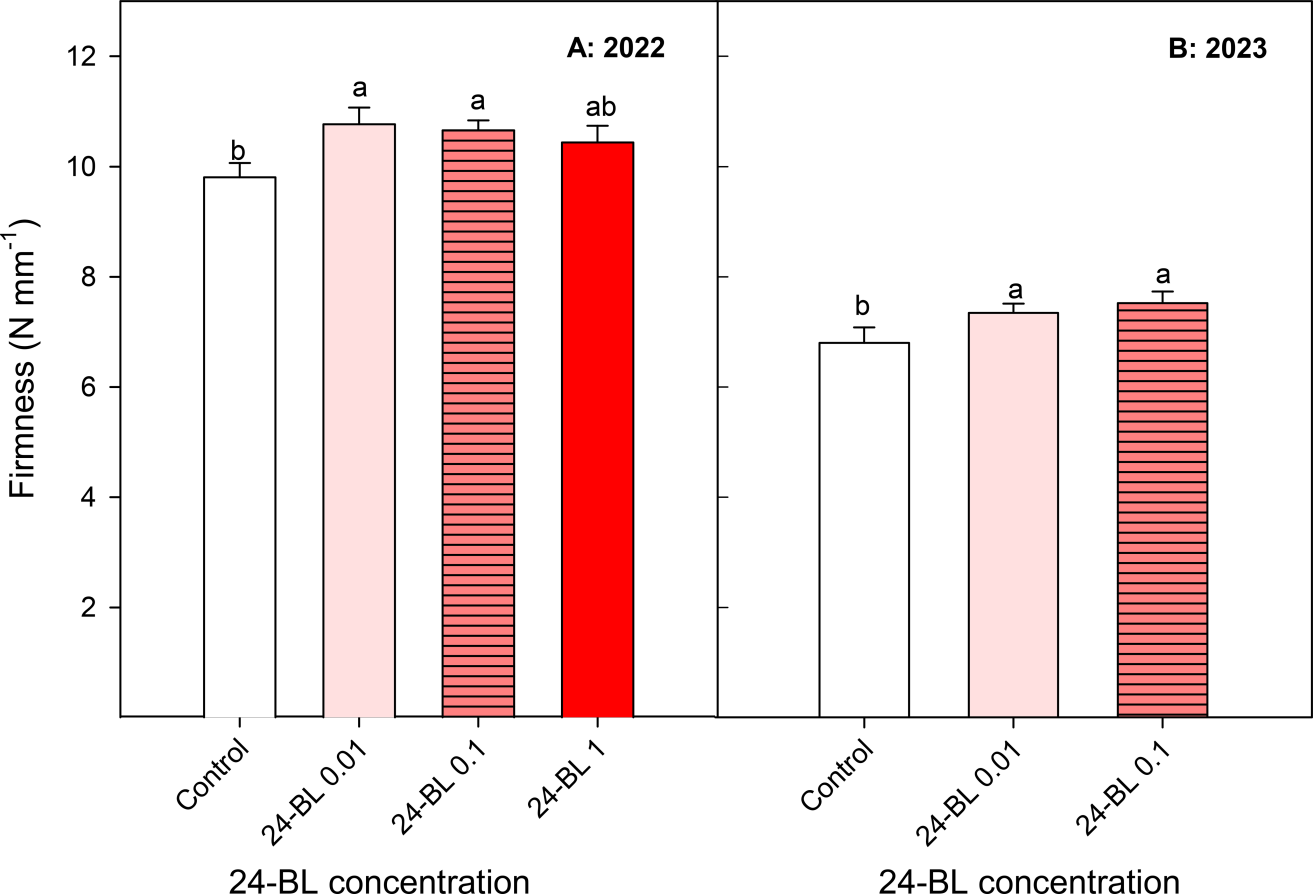
Blood orange fruit firmness (N/mm) in two different seasons, 2022 **(A)** and 2023 **(B)**, as affected by 24-epibrassinolide (24-BL) treatments at 0.01, 0.1, and 1 μM. Data are presented as the mean ± SD. Different letters show significant differences (*p* < 0.05) among treatments for each growing season.

Fruit firmness, external and internal colour, TA, and TSS are the most important quality parameters in the “Sanguinelli” blood orange cultivar in terms of consumer acceptance. In general, fruit firmness was significantly increased by the 24-BL treatments in both years, although the greatest improvements were recorded at 0.01- and 0.1-μM doses in 2022, with values of 10.77 ± 0.3 and 10.66 ± 0.18 N/mm, respectively, compared to 9.80 ± 0.26 N/mm in fruits from the control trees (**Figure 3A**). In the 2023 harvested fruits, similar increases in fruit firmness were observed as a consequence of the 24-BL treatments (**Figure 3B**).

**Figure 4:**
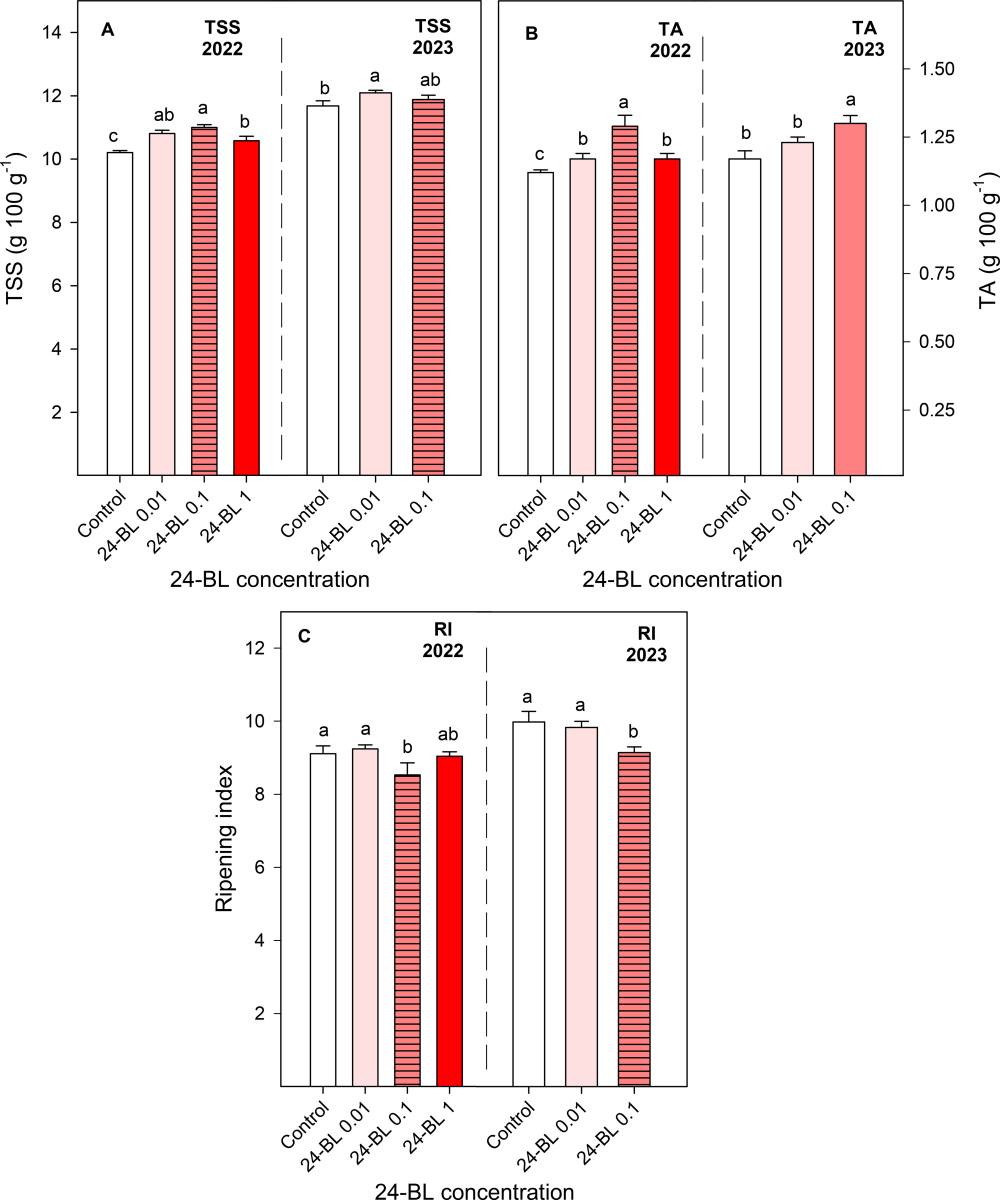
Total soluble solids (TSS); **(A)** titratable acidity (TA; **B**), and ripening index (RI; **C**) in blood orange fruit as affected by 24-epibrassinolide (24-BL) treatment*s* at 0.01, 0.1, and 1 μM. Data are presented as the mean ± SD. Different letters show significant differences (*p* < 0.05) among treatments for each year.

TSS and TA were also increased as a consequence of the 24-BL treatments in both seasons (**Figures 4A, B**). Fruits treated with a 0.01-μM dose showed significantly higher TSS of 10.81 ± 0.10 and 12.09 ± 0.08 g/100 g for the 2022 and 2023 seasons, respectively, compared to the controls (10.21 ± 0.06 and 11.68 ± 0.16 g/100 g, respectively) (**Figure 4A**). Regarding TA, fruits treated with 0.1-μM concentration showed the highest TA values, being 1.29 ± 0.04 g/100 g in the first season and 1.30 ± 0.01 g/100 g in the second season, in comparison to the controls at 1.12 ± 0.01 and 1.17 ± 0.03 g/100, respectively (**Figure 4B**). The ripening index (RI) was calculated as the TSS/TA ratio. According to this value, the preharvest treatment with 24-BL at 0.1 μM significantly reduced the RI in both seasons (**Figure 4C**). In addition, it is worth noting that TSS were higher in the 2023 season than in 2022 in fruits from both control and treated trees. This fact, together with the lower firmness in 2023 than in 2022, could indicate that the fruits in 2023 were in a little more advanced ripening stage.

**Figure 5:**
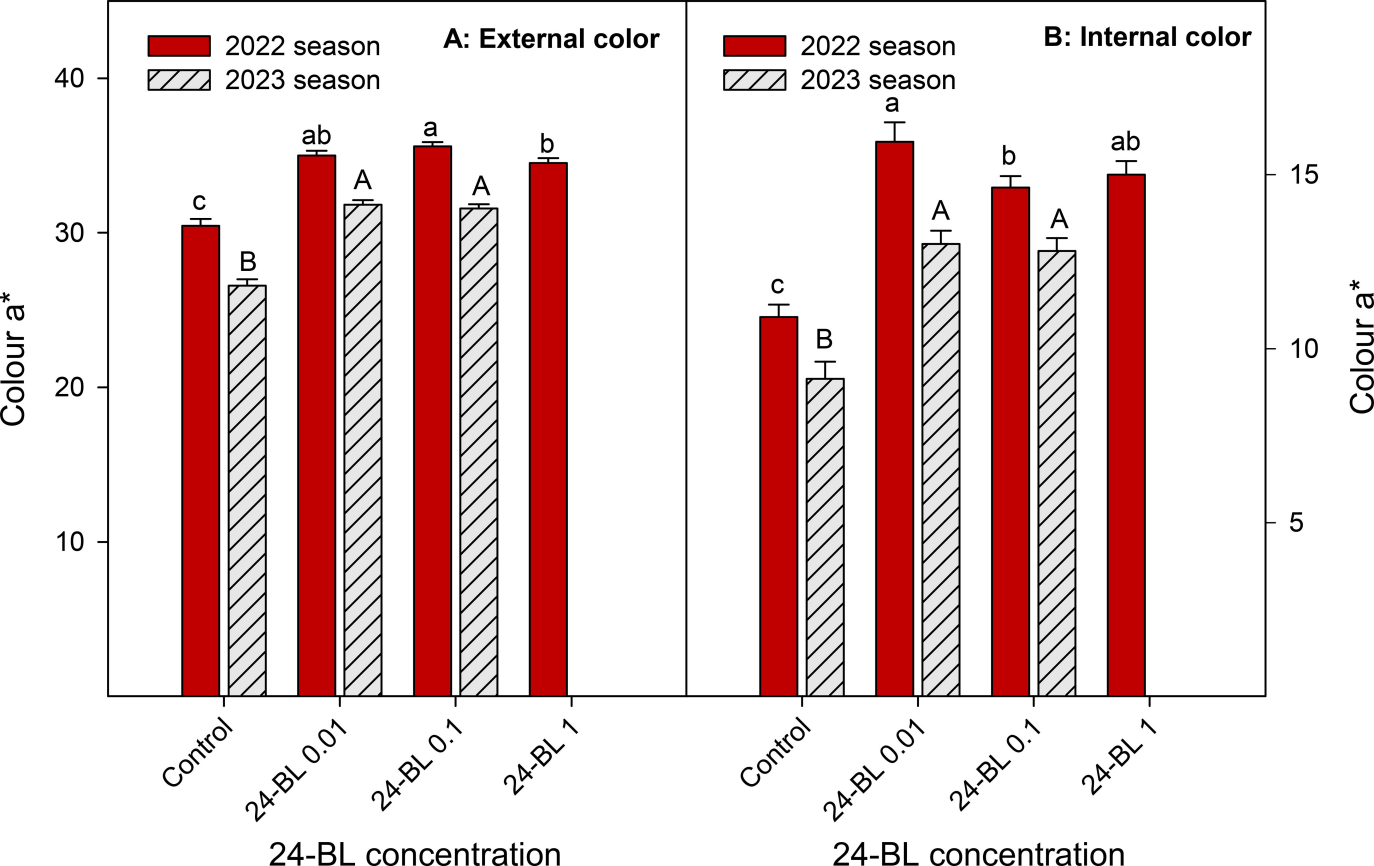
External **(A)** and internal **(B)** colour (a* coordinate) in blood orange fruit in 2022 and 2023 seasons as affected by 24-epibrassinolide (24-BL) treatments at 0.01, 0.1, and 1 μM. Data are presented as the mean ± SD. Different lowercase and capital letters show significant differences (*p* < 0.05) among treatments in 2022 and 2023 seasons, respectively.

**Figure 6:**
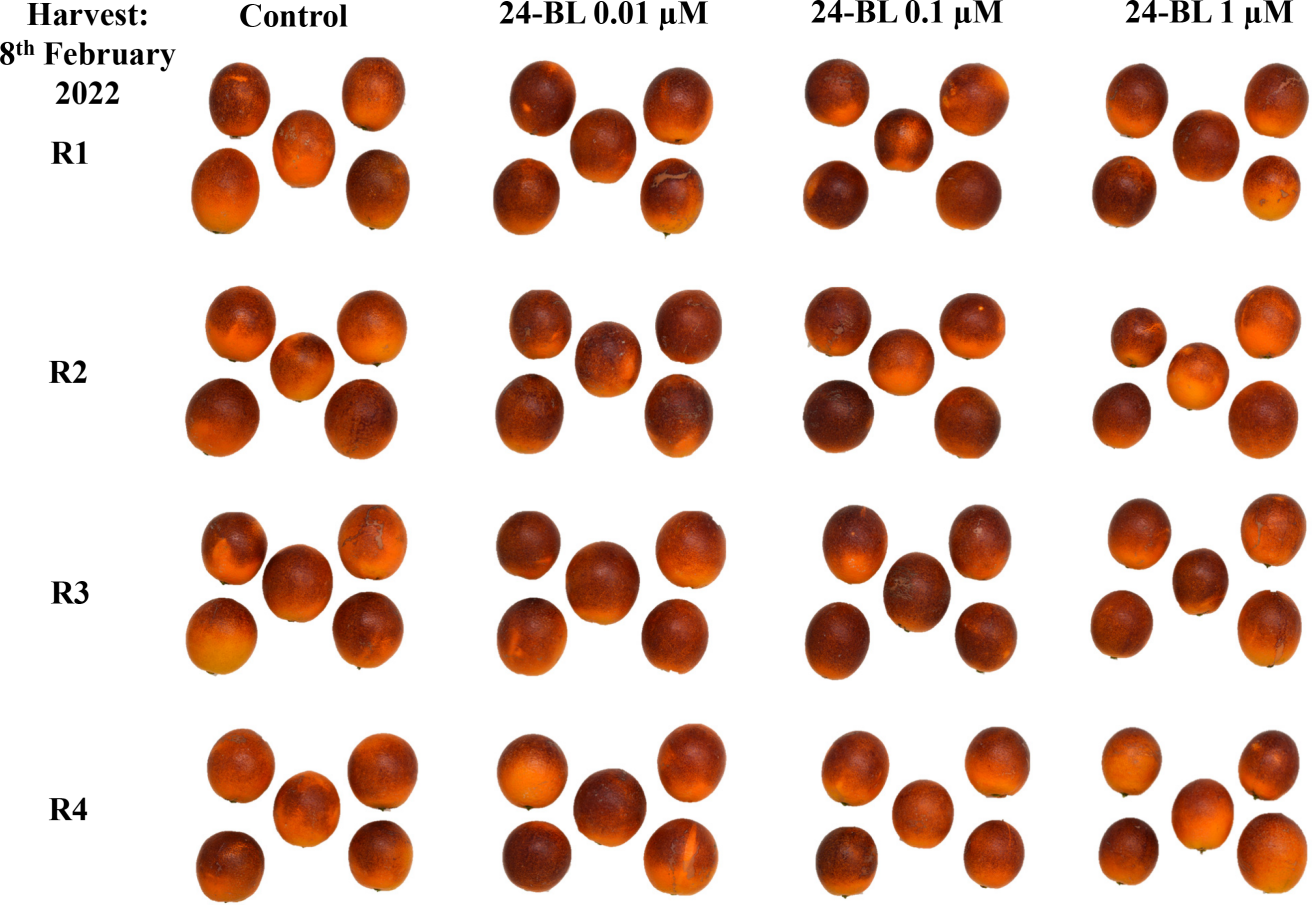
External fruit appearance of orange fruits at harvest in 2022 experiment as affected by 24-epibrassinolide (24-BL) treatments at 0.01, 0.1, and 1 μM. R1–R4 mean four replicates of five fruits.

**Figure 7:**
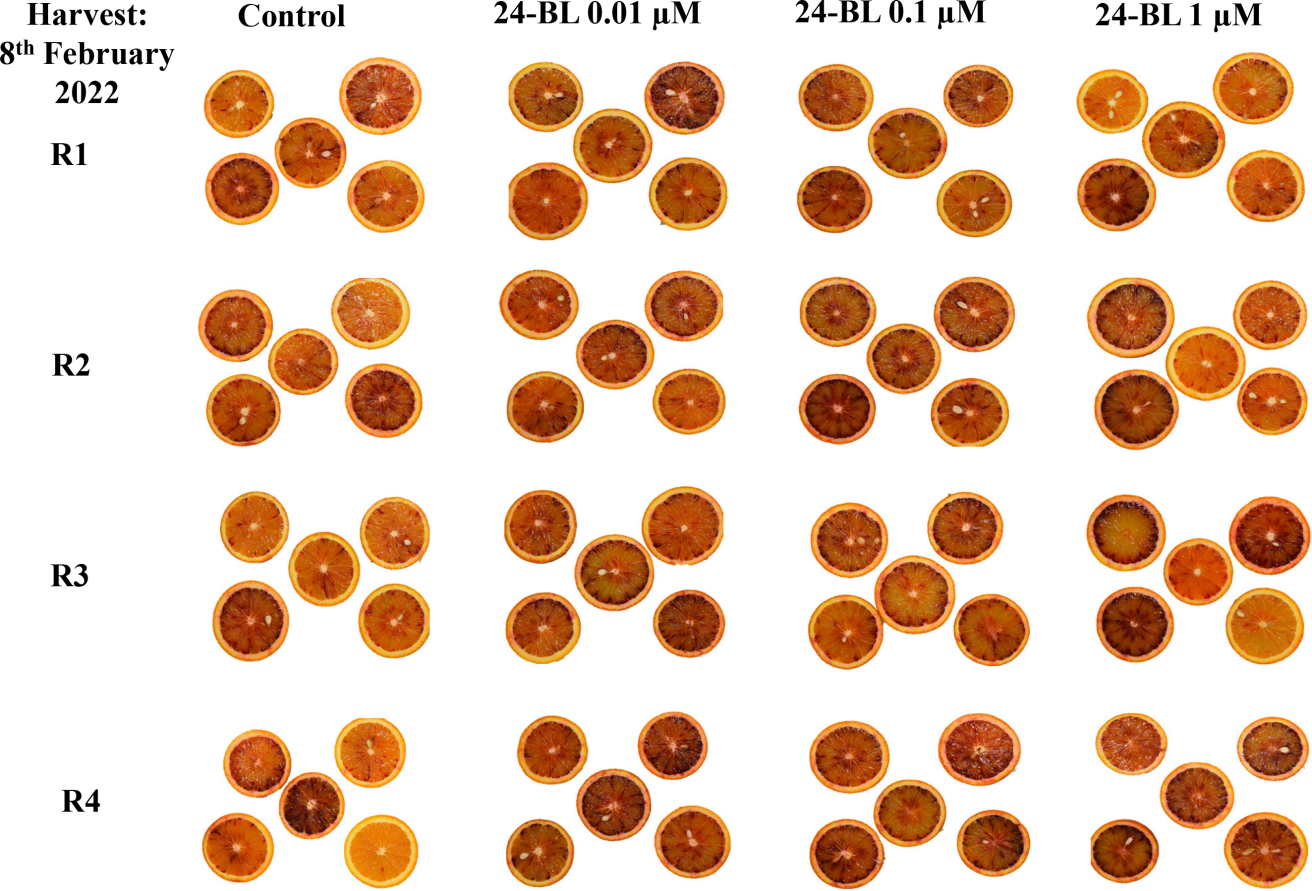
Internal fruit appearance of orange fruits at harvest in 2022 experiment as affected by 24-epibrassinolide (24-BL) treatments at 0.01, 0.1, and 1 μM. R1–R4 mean four replicates of five fruits.

External and internal colours are among the most important quality parameters in blood oranges. In both seasons, all 24-BL treatments significantly increased both external and internal colour a* (**Figure 5**). In the 2022 season, fruits from the trees treated with 0.01 μM 24-BL showed the highest a* internal value (15.95 ± 0.56), while fruits from the trees treated with a 0.1-μM dose had the highest external value (35.59 ± 0.28). Control fruits showed 10.91 ± 0.36 and 30.45 ± 0.44 colour a* values for internal and external colours, respectively (**Figure 5A**). Nevertheless, in the 2023 season, blood oranges from trees treated with 0.01-μM 24-BL had the highest values compared to the rest of the treatments, 13.01 ± 0.38 and 31.82 ± 0.29, for internal and external a* colour, respectively (**Figure 5B**). It is important to note that higher values for a* colour mean a more intense red colouration than lower values and, in turn, the 24-BL treatments led to fruits with increased red colour in either peel or flesh, as can be observed in the photographs of **Figures 6** and **7** in the 2022 experiment and **Supplementary Figures 1** and **2** in the 2023 experiment.

### 3.4 Anthocyanins in juice and flavedo

**Figure 8:**
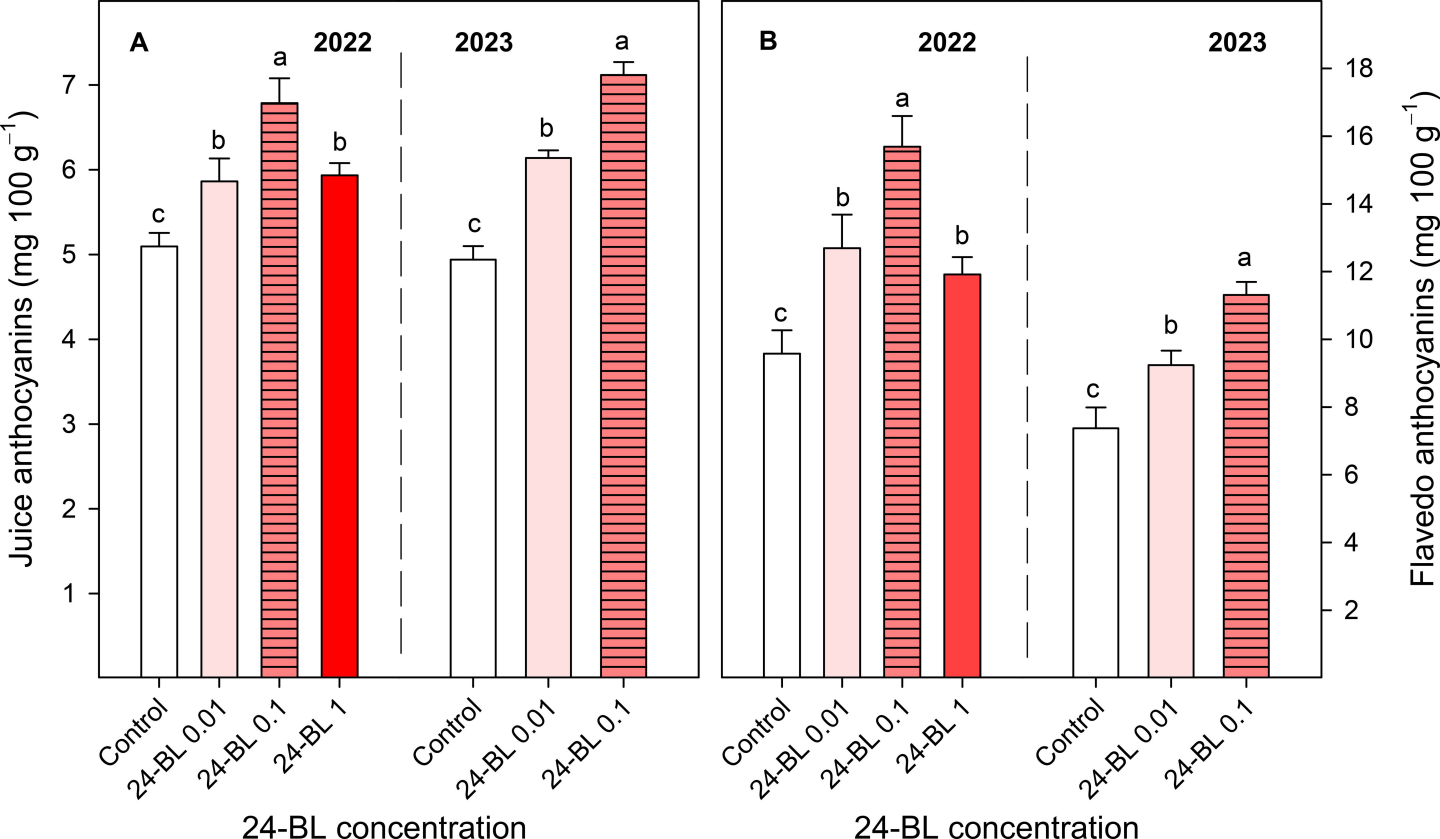
Juice and flavedo total anthocyanin contents in two different seasons, 2022 **(A)** and 2023 **(B)**, in blood orange fruit as affected by 24-epibrassinolide (24-BL) treatments at 0.01, 0.1, and 1 μM. Data are presented as the mean ± SD. Different letters show significant differences (*p* < 0.05) among treatments for each year.

Total anthocyanin content in the juice was significantly increased for all 24-BL treatments in both years compared to the controls. In the 2022 season, a 0.1-μM dose led to the highest total anthocyanin content in the juice, with values of 6.79 ± 0.29 mg/100 g, as well as in 2023 (7.12 ± 0.15 mg/100 g), while in the control fruits, 5.09 ± 0.15 and 4.94 ± 0.16 mg/100 g total anthocyanin contents were found in 2022 and 2023, respectively (**Figure 8A**). Similar effects of the 24-BL treatment were observed in the flavedo tissue. In both seasons, blood orange fruits from trees with preharvest treatments of 0.01- and 0.1-μM 24-BL showed significantly higher anthocyanin levels than the controls, the highest effects being observed for the 0.1-μM 24-BL treatment, with concentrations of 15.69 ± 0.91 and 11.31 ± 0.39 mg/100 g in the first and second seasons, respectively, while in fruits from the controls, those values were 9.57 ± 0.69 mg/100 g in the 2022 season and 7.37 ± 0.62 mg/100 g in 2023 (**Figure 6B**). Anthocyanin levels were lower in the peel of all treatments in the second season compared to the first season (**Figure 8B**).

**Figure 9:**
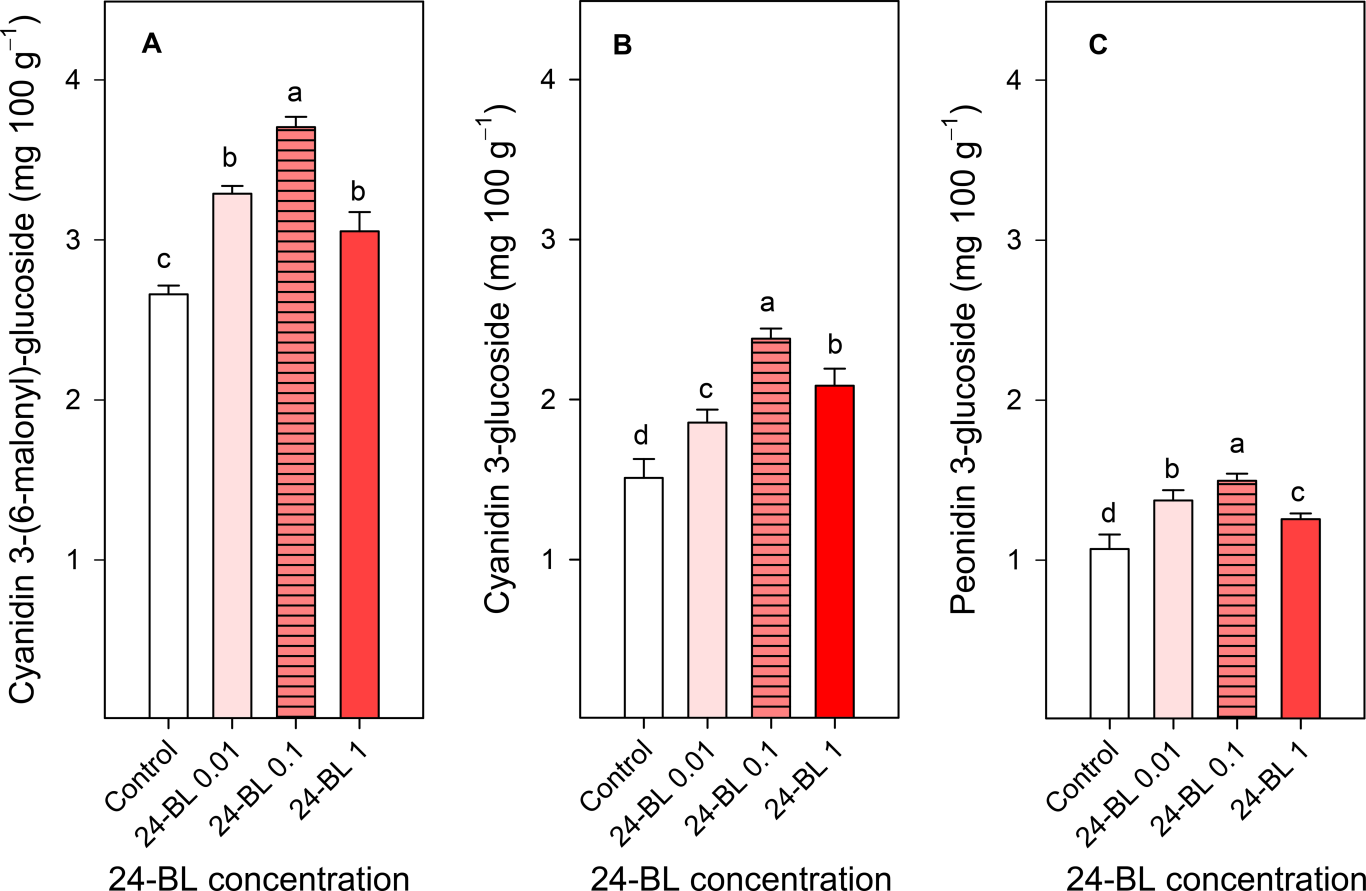
Cyanidin 3-(6-malonyl)-glucoside **(A)**, cyanidin 3-glucoside **(B)** and peonidin 3-glucoside **(C)** concentrations in 2022 in blood orange as affected by 24-epibrassinolide (24-BL) treatment at 0.01, 0.1 and 1 μM. Data are presented as the mean ±SD. Diferent letters show significant differences (*p* < 0.05) among treatments.

The increase in total anthocyanin content, as a consequence of the 24-BL treatments, was confirmed by the quantification of individual anthocyanins in 2022 since the three individual anthocyanins quantified were significantly enhanced by all 24-BL applied doses, with the highest effects being observed for 0.1-μM concentration (**Figure 9**). The major anthocyanin in blood orange juice was cyanidin 3-(6-malonyl)-glucoside, ranging from 2.65 ± 0.05 to 3.70 ± 0.06 mg/100 g in the control and 0.1-μM 24-BL preharvest treatment (**Figure 9A**), followed by cyanidin 3-glucoside, with values of 1.51 ± 0.11 and 2.38 ± 0.07 mg/100 g in the control and 0.1-μM 24-BL, respectively (**Figure 9B**); peonidin 3-glucoside was found at the lowest concentrations, from 1.06 ± 0.09 to 1.50 ± 0.04 mg/100 g in the control and 0.1-μM 24-BL, respectively (**Figure 9C**).

### 3.5 Total phenolic content in juice and flavedo

**Figure 10:**
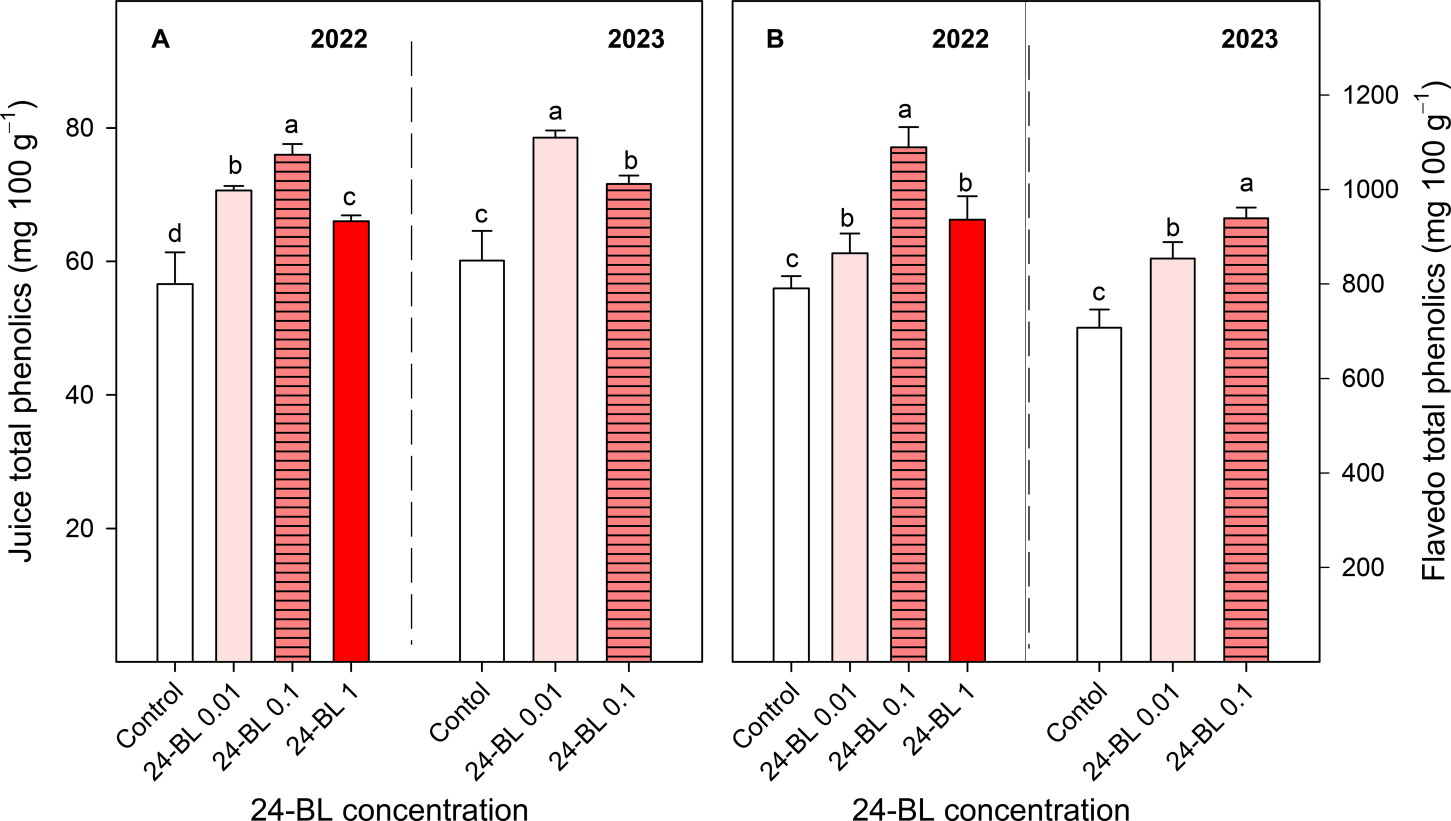
Juice **(A)** and flavedo **(B)** total phenolic content in two different seasons, 2022 and 2023, in blood orange fruit as affected by 24-epibrassinolide (24-BL) treatments at 0.01, 0.1, and 1 μM. Data are presented as the mean ± SD. Different letters show significant differences (*p* < 0.05) among treatments for each year.

Treatments with 24-BL also increased the content of total phenolics compared to fruits from the control trees in both seasons, in either the juice or flavedo (**Figures 10A, B**). For juice samples, preharvest treatment with 0.1-μM concentration led to the highest value of phenolic content (76.00 ± 1.59 mg/100 g), an increase of 34% compared to that of the controls, while in 2023, 0.01-μM 24-BL was the treatment that showed the highest value (78.53 ± 1.10 mg/100 g), 31% higher than that of the controls (**Figure 10A**). For flavedo samples, the total phenolic content was also significantly increased by the 24-BL treatments, and the highest effect was observed for a 0.1-μM dose in both seasons, with values of 1,089.59 ± 42.85 mg/100 g in the first season and 839.33 ± 22.48 mg/100 g in the second season, which were 38% and 33% higher, respectively, than in those fruits from the control trees (**Figure 10B**).

## 4 Discussion

The 24-BL treatments, at 0.01- and 0.1-μM doses, significantly increased crop yield, with an enhanced number of fruits harvested by tree (**Figure 1**). It is worth noting that these observed effects were similar for both growing seasons (11.5% and 15% increases for the 0.1-μM dose in 2022 and 2023, respectively), showing that the effects of the 24-BL treatments on increasing crop yield in blood orange trees are independent of the climatic differences that could have occurred in both years (**Supplementary Table 2**). Interestingly, the yield of commercial fruits was also increased by the 24-BL treatments, with the highest increase, 41%, being observed for the 0.1-μM treatment (**Figure 2A**). Thus, 24-BL could have a high impact on economic profit for growers since commercial fruits can be sold as blood oranges for fresh consumption, while non-commercial fruits are sold to the juice industry, and prices are higher at the fresh market than at the juice industry market. In addition, the increase in yield (kg/tree) was observed in commercial fruits with large size, showing that treatments led to larger-sized fruits compared to the controls. BRs have been reported to have different roles in plant development, including cell division, elongation, and expansion (Manghwar et al., 2022; Silva et al., 2024), which could lead to increased fruit growth and could explain the observed effects. In some previous papers, the beneficial effects of BR treatments on crop yield have been reported. For instance, 24-BL (at 0.1 mg/L) treatments in sugar apple trees, applied weekly from anthesis to harvest, increased crop yield, with enhanced fruit retention, number of harvested fruits per tree, and fruit weight and size (Mostafa and Kotb, 2018). Similar results were observed in pepper (Samira and Mansour, 2012), sweet cherry (Ahamed et al., 2021), and strawberry plants cultivated under normal conditions or under drought and saline stresses (Asghari and Rezaei-Rad, 2018; Khatoon et al., 2020; Furio et al., 2022). These effects were attributed to 24-BL’s role in increasing leaf area, chlorophyll content, and CO_2_ assimilation by inducing the synthesis of ribulose-1,5-bisphosphate carboxylase/oxygenase enzyme (Rubisco) and other enzymes of the Calvin cycle, and net photosynthesis rate, as reported by Yu et al. (2004) and Xia et al. (2009) in zucchini plants. Then, more photosynthates could be available to support fruit growth and increase the observed crop yield in blood oranges after the 24-BL treatments. However, it is important to note that 24-BL effects on increasing crop yield depend on the treatment concentration and the number and timing of the applications. In fact, the present results show that 0.01- and 0.1-μM doses were more effective than the 1-μM dose. Accordingly, Babalık et al. (2020) reported that BR treatment at a concentration of 0.2 mg/L applied at 7 days before fruits set, veraison, and 30 days after veraison was the most suitable treatment to increase the yield of the “Alphonse Lavallée” grape cultivar, in a study in which 0.2, 0.4, 0.6, and 0.8 mg/L concentrations and three different periods of application were assayed. Similarly, a 0.025-mg/L BL treatment of blood orange trees was more effective than a 0.015-mg/L treatment in increasing its leaf area and its content in chlorophyll and carbohydrate contents (Lateef et al., 2023).

The increased temperatures in the southeast of Spain, in the present scenario of climate change, have led to a decline in blood orange quality with poor red colouration since high temperature differences between day and night are required for the synthesis of anthocyanins (Lo Piero, 2015). Several strategies to increase red colouration in blood orange fruits have been developed in recent years. For instance, Chen et al. (2022) showed that a combined treatment of ethephon, at 2 mg/L, with a temperature of 8°C was the most effective method to increase anthocyanin accumulation in the “Tarocco” cultivar. Other researchers have reported the use of cold temperatures (3°C–4°C) as a useful postharvest tool to increase anthocyanin accumulation in blood oranges (Pannitteri et al., 2017; Crifò et al., 2011). In addition, sorbitol, methyl jasmonate, or thiabendazole applied as preharvest treatments led to the improvement of the red colour in blood orange fruits (Guirao et al., 2025; Vithana et al., 2024; Schirra et al., 2022). However, to the best of our knowledge, this is the first evidence showing the efficacy of 24-BL applied as a foliar spray preharvest treatment in increasing the red colour in the flavedo and flesh of blood oranges. In fact, a significant increase in external and internal a* colour was observed in all 24-BL-treated fruits compared to the controls (**Figure 5**) in both seasons, showing a deeper red colour, since a higher value of a* means a higher red colouration, as can be observed in the photographs in **Figures 6** and **7**, respectively, for the 2022 experiment, and in **Supplementary Figures 1** and **2**, for the 2023 experiment. Thus, 24-BL could be a useful tool with agronomic practical application since it is recognised as a non-toxic, eco-friendly, and naturally occurring compound (Ali, 2017; EPSA, 2020). However, it is worth noting that blood oranges had deeper flavedo red colour and anthocyanin content in 2022 than in 2023 (**Figures 5** and **8B**), which was related with the higher number of hours below 5°C occurring in November, December, and January in the 2021–2022 experiment than in the 2022–2023 experiment (**Supplementary Table 2**).

The highest effects of the 24-BL treatments in increasing total anthocyanin content in the flavedo and juice were found for the 0.1-μM dose (**Figure 8**). Similar effects of preharvest BR treatments on increasing red colour and anthocyanin content have been reported in other red fruit species, such as table grapes (Xu et al., 2015a; 2015b; Babalık et al., 2020) and strawberries (Ayub et al., 2018; Furio et al., 2022). These reports provide evidence on the role of BRs in anthocyanin biosynthesis in non-climacteric fruit species, in spite of the fact that abscisic acid was the first claimed hormone regulating this process (Kou et al., 2021). Accordingly, postharvest treatment by BR increased anthocyanin content in segments of blood oranges cv. “Sanguinello” during storage (Habibi et al., 2021), which was attributed to the enhanced activity in phenylalanine ammonia-lyase (PAL), the first enzyme involved in the phenylpropanoid biosynthesis pathway, as well as chalcone synthase and chalcone isomerase. Additionally, one study conducted on red grapes (Cabernet Sauvignon) claimed that the exogenous application of BRs can enhance the expression of other structural genes encoding for flavanone-3-hydroxylase, flavonoid-3′-hydroxylase, flavonoid-3′, 5′-hydroxylase, and dihydroflavonol-4-reductase as well as *MYBA1* and *MYBA2* transcription factors related to the phenylpropanoid pathway (Luan et al., 2013). Apart from that, BRs contributed to increasing anthocyanin accumulation by direct binding of the brassinazole-resistant (BZR 1) transcription factor to the MYB transcription factor in *Arabidopsis thaliana* (Lee et al., 2024). In concordance with these results, a recent study has shown that the upregulated differentially expressed proteins (DEPs) annotated using the Kyoto encyclopedia of genes and genomes (KEGG), gene ontology (GO), and eukaryotic orthologous groups/clusters of orthologous groups (KOG/COG) databases were enriched in the biosynthesis and metabolism of BRs, which could mean that BRs are involved in the regulation of cold-induced anthocyanin accumulation in blood oranges (Chen et al., 2025).

Individual anthocyanins were also increased by the 24-BL treatments, especially with 0.1-μM concentration (**Figure 9**). The major anthocyanin in blood orange fruit was cyanidin 3-(6″-malonylglucoside), followed by cyanidin 3-glucoside and peonidin 3-glucoside, according to previous reports on “Sanguinelli” and other blood orange cultivars, although their concentrations depend on the studied cultivar (Ballistreri et al., 2019; Habibi et al., 2022a). Total phenolic content was also increased by the 24-BL treatments in both the flavedo and juice, and the greatest improvements were recorded at 0.1 μM for both assayed seasons (**Figure 10**). This effect has been attributed to an increase in the activity of PAL, as commented above, or to the reduced activity of two phenolic oxidant enzymes, polyphenol oxidase (PPO) and peroxidase (POD) (Gao et al., 2016; Xu et al., 2015a). Thus, preharvest treatments with 24-BL could meet consumer demands since one of the most important quality parameters of blood orange fruits valued by consumers is their red colour (Simons et al., 2019). In addition, the enhanced phenolic content in blood oranges from 24-BL-treated trees could improve the health benefits of blood orange consumption since anthocyanins, flavonoids, and other phenolic compounds are recognised phytochemicals that exhibit important antioxidant activity and help in combatting diseases such as diabetes, Alzheimer’s, or cancer (Raghavan and Gurunathan, 2021; Chen et al., 2024).

Other important quality parameters in fresh fruits are firmness, TSS, and TA, which show important differences among fruit species and cultivars and are also affected by environmental and agronomical factors, although, in general, TSS increase with maturation, while reductions occur in firmness and TA (Valero and Serrano, 2010; García-Pastor et al., 2024). The present results show an increase in firmness, TSS, and TA in blood oranges for all 24-BL treatments and seasons, and the 0.1-μM dose led to a significant decrease in RI compared to the controls. Thus, the effect of 24-BL on modulating blood oranges on-tree maturation does not follow a general trend for all parameters since the increase in TSS could indicate acceleration of maturation, while the higher values of firmness and TA could show a delay in this process. BR treatment of strawberries led to fruits with better firmness than fruits from control plants at harvest (Ayub et al., 2018), as well as BR treatments in “Red Delicious” apples (Wang et al., 2024), showing an effect in delaying the on-tree ripening process. The higher firmness of 24-BL-treated fruit trees compared to the controls (**Figure 3**) could reveal a positive effect of the 24-BL treatments on the shelf life of blood oranges during storage. This increase in firmness can be explained by the role of BRs in promoting cell expansion through BR-regulated expression of genes involved in cell wall modifications, ion and water transport, and cytoskeleton rearrangements (Clouse, 2011). Higher values of TSS and TA were also observed in “Thompson” seedless table grapes at harvest as a consequence of BR treatments (Asghari and Rezaei-Rad, 2018). However, Khatoon et al. (2020) reported an increased ripening index (TSS/TA ratio) in strawberries from BR-treated plants, in agreement with the finding of Mostafa and Kotb (2018) on sugar apples. For postharvest BR treatments, acceleration of the ripening process has been reported in persimmons (He et al., 2018) and tomatoes (Zhu et al., 2015), due to increased ethylene production. Despite these different effects on each particular maturation stage and quality traits, in general, improvements of fruit quality have been claimed after BR preharvest or postharvest treatments in several fruit species, according to the present results on blood orange fruits.

## 5 Conclusions

The present results provide evidence regarding the beneficial effects of the preharvest 24-BL treatments, applied as a foliar spray at three key points of fruit development, on improving crop yield and fruit quality properties at harvest of “Sanguinelli” blood oranges. The most important effects were observed for 0.1-μM 24-BL concentration, and the most important improved quality parameter was red colour, in the flavedo and juice, with enhanced total and individual anthocyanin concentrations. The increase in total and commercial fruit yield could lead to enhanced growers’ incomes since only blood orange fruits with more than 25% of their surface with red colour can be commercialised as blood oranges, and they reach higher prices than non-commercial ones. Moreover, consumer acceptance could increase since red colour is the most important parameter valued by consumers in blood oranges, apart from enhanced health benefits by increased anthocyanins and total phenolics. Nevertheless, more research is needed to find out if reducing the number of treatments could reach similar results and be more cost-efficient. In addition, applying metabolomic analysis to detect additional nutritional components could provide valuable information related to the fruit quality of blood oranges and the underlying physiological mechanisms involved; therefore, it deserves further research.

## Data Availability

The original contributions presented in the study are included in the article/**Supplementary Material**. Further inquiries can be directed to the corresponding author.
