# Supplementary material for: Brassinosteroid preharvest treatments as a useful tool to increase crop yield and red colour in blood orange fruits
Source: Front Plant Sci. 2025 Sep 2;16:1654517. doi: 10.3389/fpls.2025.1654517 (PMC12436118; doi:10.3389/fpls.2025.1654517)
Supplement: Supplementary file 1 [file DataSheet1.docx]

Supplementary Material.

# Supplementary Figures and Tables

## Supplementary Figures


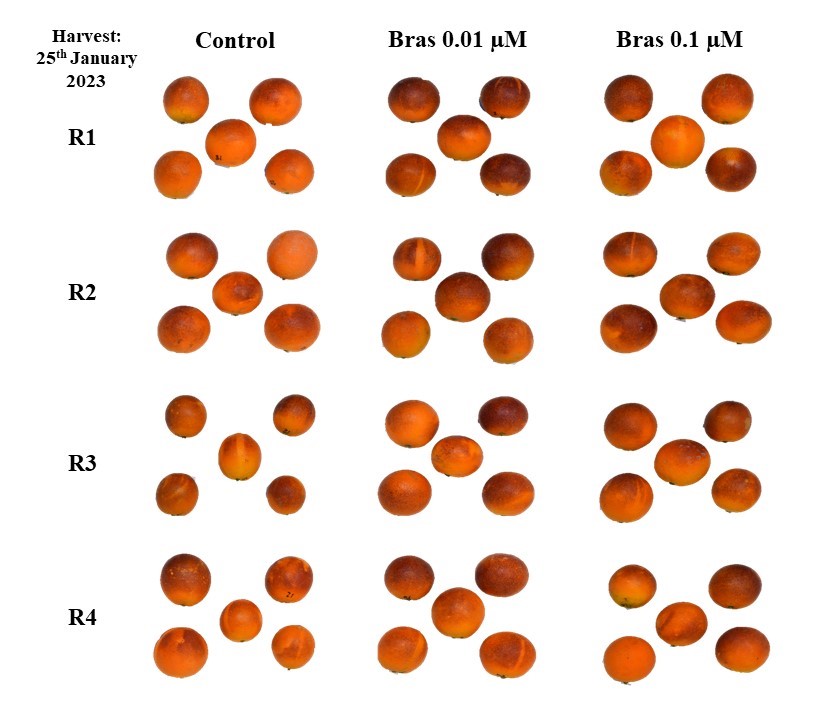


**Supplementary Figure 1.** External fruit appearance of orange fruit at harvest in 2023 experiment as affected by 24-epibrassinolide (Bras) treatment*s* at 0.01 and 0.1 µM. R1-R4 mean four replicates of 5 fruits.


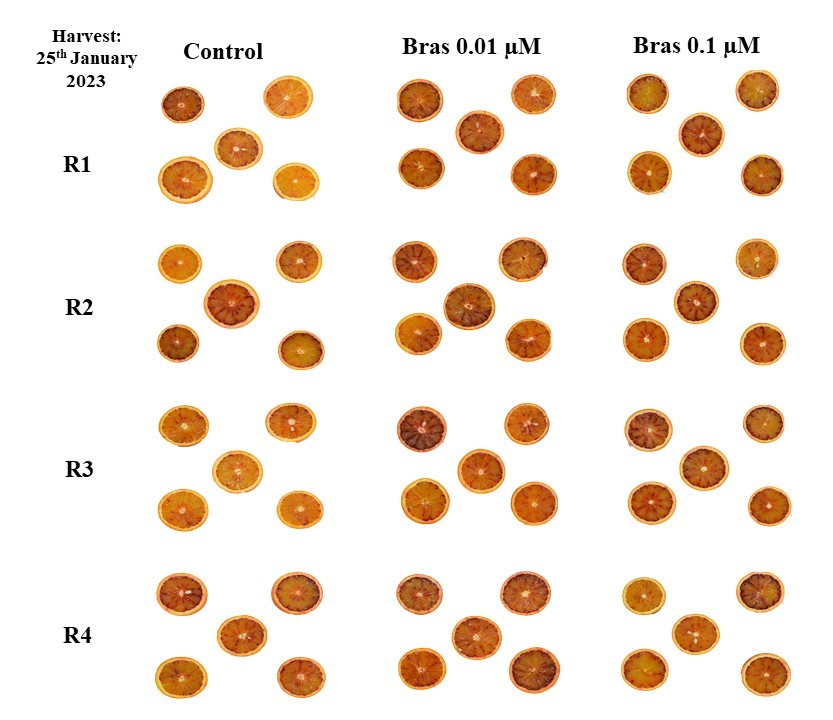


**Supplementary Figure 2.** Internal fruit appearance of orange fruit at harvest in 2023 experiment as affected by 24-epibrassinolide (Bras) treatment*s* at 0.01 and 0.1 µM. R1-R4 mean four replicates of 5 fruits.

## Supplementary Tables

**Supplementary Table 1.** Average field temperatures: Maxim (T_max_), middle (T_mid_), minimum (T_min_) and number of hours in which blood oranges have been exposed at 5 °C or less (H < 5 °C) before harvesting blood orange fruits in two seasons (2021-2022 and 2022-2023).

|  | **November** | | **December** | | **January** | |
| --- | --- | --- | --- | --- | --- | --- |
|  | **2021** | **2022** | **2021** | **2022** | **2022** | **2023** |
| **T_max_ (°C)** | 16.95 | 20.18 | 16.36 | 17.18 | 16.24 | 17.72 |
| **T_min_ (°C)** | 8.01 | 10.44 | 7.99 | 7.95 | 5.79 | 6.56 |
| **T_mid_(°C)** | 11.86 | 14.78 | 11.74 | 11.81 | 10.42 | 11.38 |
| **Hours at < 5 °C** | 12 | 5 | 24 | 14 | 97 | 68 |
