# Supplementary figures and images for: Brassinosteroid preharvest treatments as a useful tool to increase crop yield and red colour in blood orange fruits
Source: Front Plant Sci. 2025 Sep 2;16:1654517. doi: 10.3389/fpls.2025.1654517 (PMC12436118; doi:10.3389/fpls.2025.1654517)

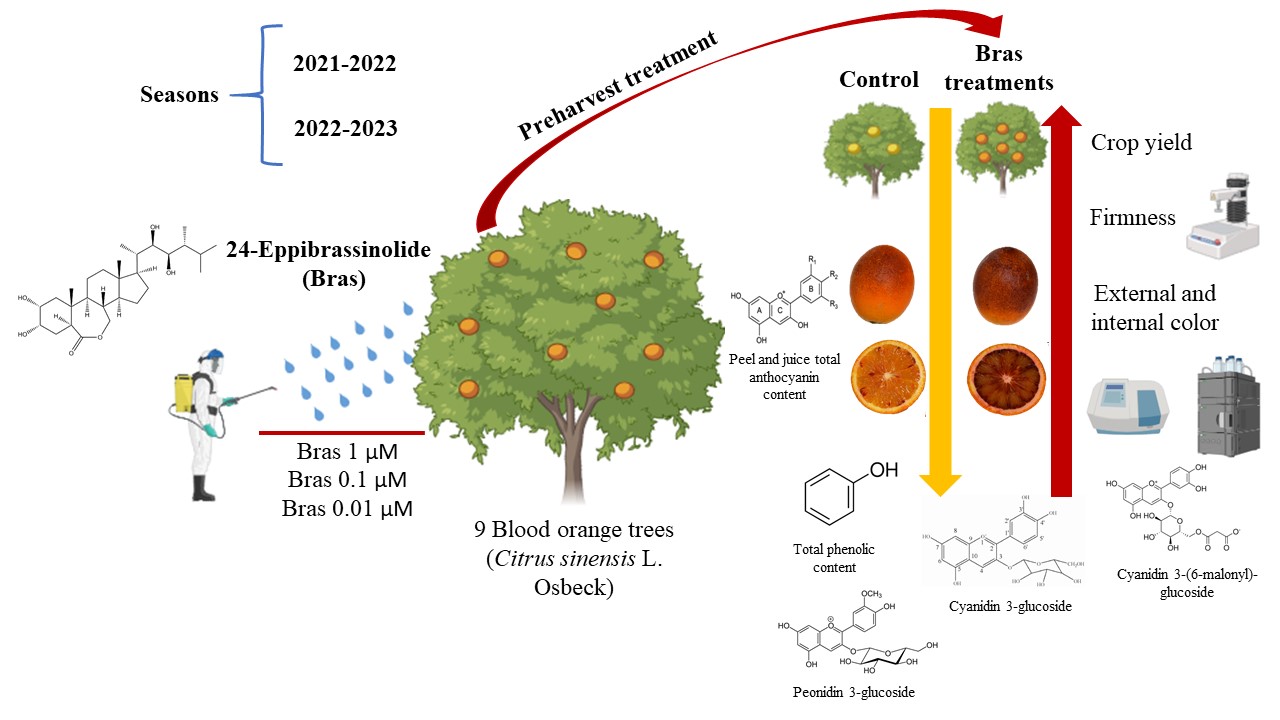

Supplement: Supplementary file 2 [file Image1.jpeg]
